# Markerless Rat Behavior Quantification With Cascade Neural Network

**DOI:** 10.3389/fnbot.2020.570313

**Published:** 2020-10-27

**Authors:** Tianlei Jin, Feng Duan, Zhenyu Yang, Shifan Yin, Xuyi Chen, Yu Liu, Qingyu Yao, Fengzeng Jian

**Affiliations:** ^1^Department of Artificial Intelligence, Nankai University, Tianjin, China; ^2^Characteristic Medical Center of the Chinese People's Armed Police Force, Tianjin, China; ^3^Key Laboratory of Exercise and Health Sciences of Ministry of Education, Shanghai University of Sport, Shanghai, China; ^4^Department of Neurosurgery, China International Neurological Institute, Xuanwu Hospital, Capital Medical University, Beijing, China; ^5^Research Center of Spine and Spinal Cord, Beijing Institute for Brain Disorders, Capital Medical University, Beijing, China

**Keywords:** markerless observation method, rat landmark points estimation, rat joint motion, behavior quantification, cascade neural network

## Abstract

Quantifying rat behavior through video surveillance is crucial for medicine, neuroscience, and other fields. In this paper, we focus on the challenging problem of estimating landmark points, such as the rat's eyes and joints, only with image processing and quantify the motion behavior of the rat. Firstly, we placed the rat on a special running machine and used a high frame rate camera to capture its motion. Secondly, we designed the cascade convolution network (CCN) and cascade hourglass network (CHN), which are two structures to extract features of the images. Three coordinate calculation methods—fully connected regression (FCR), heatmap maximum position (HMP), and heatmap integral regression (HIR)—were used to locate the coordinates of the landmark points. Thirdly, through a strict normalized evaluation criterion, we analyzed the accuracy of the different structures and coordinate calculation methods for rat landmark point estimation in various feature map sizes. The results demonstrated that the CCN structure with the HIR method achieved the highest estimation accuracy of 75%, which is sufficient to accurately track and quantify rat joint motion.

## Introduction

Rats, which are genetically similar to humans with low feeding costs, have been widely used in research of neuroscience, medicine, the social sciences, and other fields (Scaglione et al., 2014; Chan et al., 2017; Zhang et al., 2017). Researchers often verify the reliability of drugs or treatments by observing the behavior of rats. For example, studies have been carried out on the relationship between limb movement and the brain in rats (Slutzky et al., 2011; Rigosa et al., 2015) as well as the effects of electrical stimulation on neural regeneration by observing spinal cord-injured rats (Joo et al., 2018). Determination of how to best observe and analyze rat behavior has long constituted a major research focus. In the past few decades, the observation method of rats' behavior has been continually developed.

In initial studies, in order to confirm experimental results by observing the behavior of rats, some researchers proposed the open field (Walsh and Cummins, 1976) and water maze (Morris, 1984) experiments. With advancements in image processing technology, many new methods for rat behavior observation were developed, such as HomeCageScan, EthoVision, and MiceProfiler (De Chaumont et al., 2012). However, these techniques are highly sensitive to the features of color and texture and are limited by the background environment, thus are not robust in long-term observation tasks. With the deepening of research, investigations of rat behavior observation have become more detailed, and a higher level of robustness is required (Weissbrod et al., 2013; Wenger et al., 2014; Nanjappa et al., 2015). Therefore, invasive sensors or markers are used to acquire more robust behavior observations for neuroscience or social science research (Weissbrod et al., 2013; Wenger et al., 2014). These methods, however, necessitate complex surgery or special markers to achieve the desired results (Burgos-Artizzu et al., 2012; Ohayon et al., 2013; Eftaxiopoulou et al., 2014; Maghsoudi et al., 2017). In the past 2 years, the observation of rat behavior based on deep neural networks has greatly improved the robustness of the observation results without the need for invasive sensors or markers (Mathis et al., 2018; Jin and Duan, 2019). Although these rat behavior observation methods are all macroscopic, which solves the problem of the rat's location and the rat's behavior at a specific time point, they do not reveal how the rat is moving. As a consequence, in this paper, we focus on rat landmark estimation to quantify joint motion and conduct locomotor kinematic analysis.

Locomotor kinematic analysis can quantitatively evaluate the locomotor recovery of the rat, which offers major potential applications for disease research, such as spinal cord injury, Parkinson's disease, traumatic brain injury, cognitive impairment, and other movement disorders (Ilha et al., 2006; Schang et al., 2011; Wenger et al., 2014). However, there are few features of rat joint points, and traditional quantitative methods of joint motion frequently need to be marked in advance. Taking the Vicon system (Schlagenhauf et al., 2018) as an example, experimental rats have to be transferred into a specialized laboratory, and researchers must spend time adjusting numerous parameters. Although the Vicon system is very reliable, it requires complex construction and preprocessing. Fortunately, with the development of deep learning in the field of computer vision, researchers can now utilize deep neural networks to extract more abundant features from images, which can be employed to detect or estimate image contents including landmarks. Specifically, for the task of human pose estimation, investigators have designed various neural network structures to automatically extract deep image features (Newell et al., 2016; Wei et al., 2016; Fang et al., 2017; He et al., 2017), and many coordinate calculation methods are utilized to locate human joints (Toshev and Szegedy, 2014; Carreira et al., 2016; Chu et al., 2017; Nibali et al., 2018) in the image. These solutions inspired us to study rat landmark point estimation and quantify rat joint motion without using any markers.

Our approach is based on the detection of the rat's position and follows the paradigm of human pose estimation. Specifically, we designed a special running machine for the rat and used a frame rate camera to capture its motion. For the estimation process, we used our previous work on rat observation to detect the rat's position (Jin and Duan, 2019). Moreover, in order to discern the landmark points including the eyes and joints, we designed two different cascade neural networks with three different coordinate calculation methods. Finally, under a strict evaluation criterion, 75% estimation accuracy was achieved. When the rat is moving on the special running machine, our approach realized the trajectory, and quantification of rat joint motion.

In summary, the main contributions of our work are 3-fold.

- We designed two neural network structures with three coordinate calculation methods to estimate landmark points and verify the effectiveness of these structures and methods.
- We only used image processing to estimate the landmark points and realize quantification of rat joint motion. To the best of the authors' knowledge, this is the first study to quantify the motion of rat joints without using invasive sensors or markers.
- We proposed a normalized evaluation criterion to evaluate different network structures and coordinate calculation methods reasonably, which can provide a useful reference for related research.

## Materials and Methods

### Neural Network Structure

**Figure 1:**
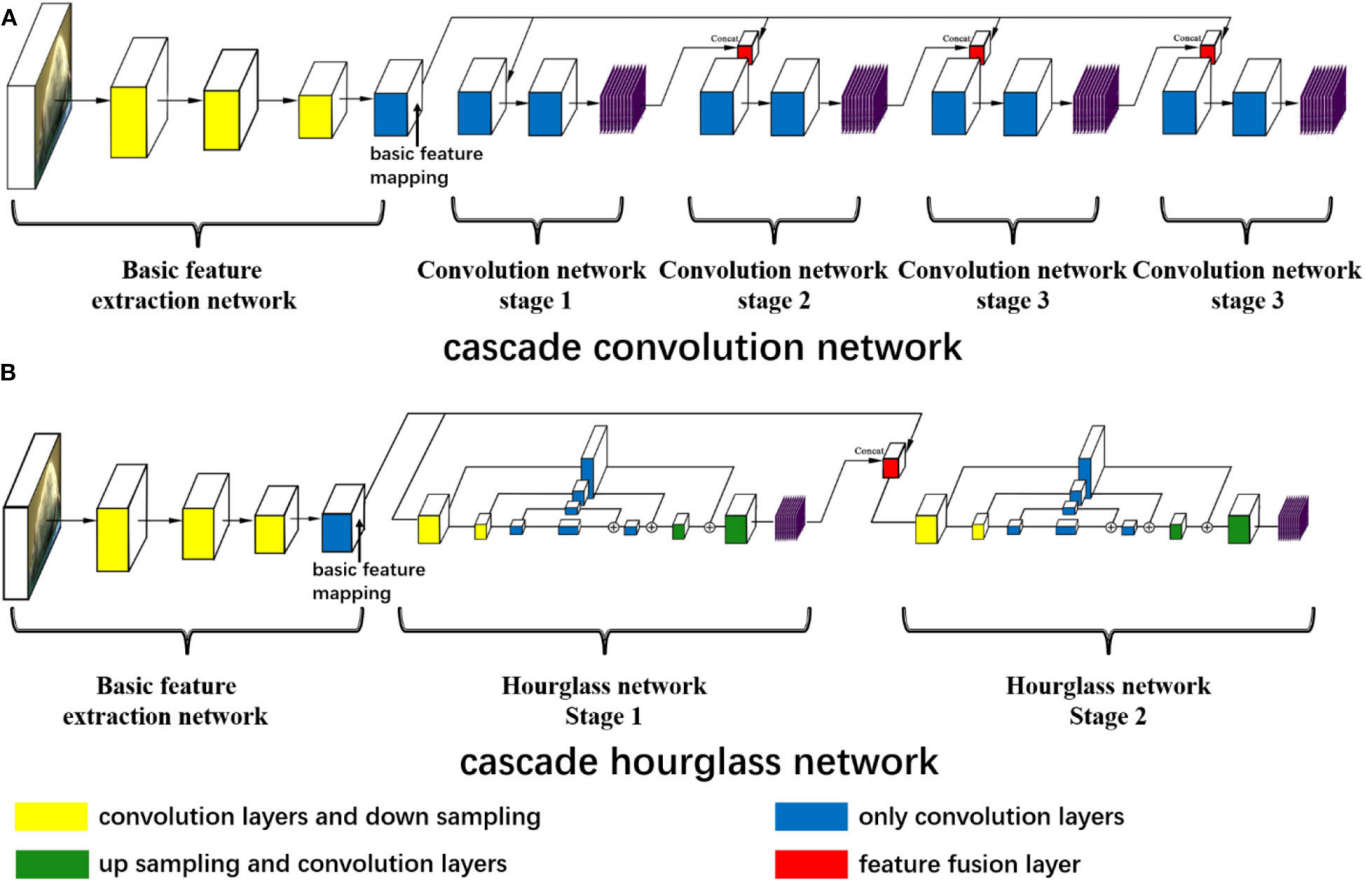
The neural network structures. **(A)** The cascade convolution network (CCN) structure and **(B)** the cascade hourglass network (CHN) structure. **(A,B)** Both have the same basic feature extraction network including 11 convolution and three downsampling layers to get the basic feature mapping. In **(A)**, there are four stages of the cascade convolution network, the basic feature mapping concatenates with the previous stage prediction by the feature fusion layer, and the prediction outputs of each stage are obtained through multiple convolution layers. In **(B)**, there is a two-stage cascade hourglass structure, and each hourglass network has the processes of downsampling, upsampling, and residual structure of convolution. Each stage has its own prediction outputs.

The landmark estimation of the rat is very similar to that of humans, and thus we refer to two well-known network structures in human pose estimation: convolutional pose machines (Wei et al., 2016) and hourglass networks (Newell et al., 2016). But we redesign the two network structures to explore the influence of network structure on estimation. As shown in Figure 1, both network structures possess the same basic feature extraction network. For the basic feature extraction network, red green blue (RGB) three-channel images are input and convoluted by two consecutive convolution layers and then the size is reduced by the following downsampling layer. At this time, the size of the feature map is half of the input image, but the channel increases. Subsequently, these two network structures with two consecutive convolution layers and one downsampling layer are utilized to reduce the feature map size and increase the channel. Therefore, the feature map size is one-eighth of the input image size. At the end of the basic feature extraction network, five continuous convolution networks without changing the feature map size are used to obtain the basic feature mapping.

After achieving the basic feature mapping, we design the first cascade convolution network (CCN) structure for further feature extraction and prediction. For the CCN structure, as shown in Figure 1A, there are four convolution stages. The first stage only receives the basic feature mapping and then uses the deep convolution network consisting of nine convolution layers to make the feature transformation and predict the first stage outputs. In the subsequent three stages, we fuse the basic feature mapping and the previous stage prediction outputs with the concat function, and nine convolution layers are also used to predict the current stage outputs. In this way, each prediction is related to the previous prediction, which is proven to augment prediction accuracy (Wei et al., 2016).

At the same time, we design the second cascade hourglass network (CHN) structure by drawing on the hourglass network (Newell et al., 2016). For our CHN structure, as shown in Figure 1B, there are two hourglass stages, of which only has two times downsampling to reduce the feature map size and two times upsampling to recover the size. Downsampling is achieved by maximum pooling layer, while upsampling is achieved by the nearest neighbor method. In order to avoid excessive feature loss during sampling, the residual structures are used for feature connections. Similar to the CCN structure, in the CHN structure, the first stage only receives the basic feature mapping, but the second stage fuses the first stage outputs and basic feature mapping to obtain the final prediction.

### Coordinate Calculation Methods

In order to improve the accuracy of landmark estimation, we designed three completely different landmark point coordinate calculation methods. These calculations methods can be used to estimate the coordinates of landmark points at each stage prediction and train neural network with the corresponding loss function.

Firstly, the same as in certain human posture estimation tasks (Toshev and Szegedy, 2014; Carreira et al., 2016), the coordinates of landmark points can be directly regressed by the fully connected network, termed the fully connected regression (FCR) method in this paper. We reshape each prediction output used for estimating the landmark to reduce the dimension. Then, we use a fully connected layer to estimate the two-dimensional coordinates of the rat's landmark points directly. Regarding the loss calculation function, we use the smooth L1 loss (Girshick, 2015), as shown in Formula (1), to calculate the error between the label landmark point and the estimated landmark point. When the estimation landmark is close to the label, L2 loss has a larger gradient and converges more rapidly. When the distance between the estimated landmark and the label is large, L1 loss can prevent the gradient explosion and reduce the impact of outliers.

(1) $$\begin{array}{l} {\text{smooth}}_{\text{L}1}\left(x\right)=\{\begin{array}{cc} 0.5x^{2} & \text{if }|x|<1\\ |x|-0.5 & |x|\geq 1 \end{array} \end{array}$$

In Formula (1), *x* is the L1 loss between the label point and the estimated point.

**Figure 2:**
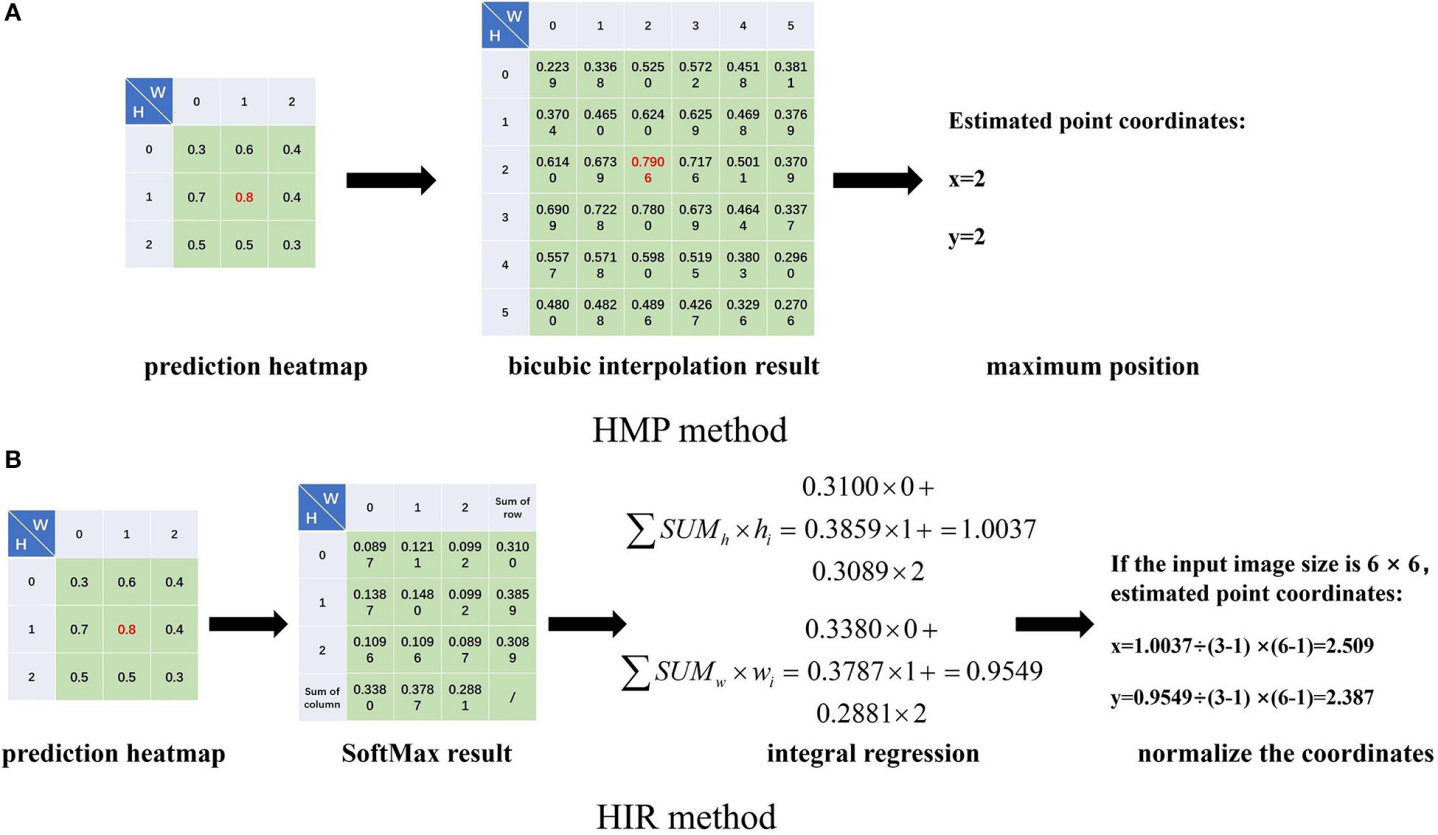
The heatmap maximum position (HMP) **(A)** method and heatmap integral regression (HIR) method introduction. We obtain the prediction heatmap from the prediction outputs of the neural networks at each stage. For the HMP method **(A)**, we use bicubic interpolation to restore the input image size, for example from 3 × 3 to 6 × 6, and we take the position of the maximum value as the coordinates of the estimated landmark point. For the HIR method **(B)**, we perform the SoftMax operation on the heatmap and sum the rows and columns, respectively. Then, we integrate the sum results and index value multiplication. Finally, the coordinates of the estimated landmark point are adjusted by normalization.

Secondly, the estimation of landmark points can be regarded as a probability problem (Burgos-Artizzu et al., 2012; Ohayon et al., 2013; Chu et al., 2016; Yang et al., 2016). We can determine the label coordinates of the landmark points in the label. Here, we assume that the abscissa and ordinate of a point are independent of each other, and then we utilize an approximate two-dimensional Gaussian function, as shown in Formula (2), to produce a Gaussian probability heatmap for each landmark point. Correspondingly, for each landmark point estimation, we also estimate a probabilistic heatmap. When we estimate the coordinates of landmarks, we can use bicubic interpolation to restore the estimated heatmap to the size of the input image and select the positions of the maximum probability value as the coordinates of the landmark point, shown in Figure 2A. We term this the heatmap maximum position (HMP) method. As for the loss function, the operation to obtain the positions of the maximum probability value is also named argmax, which is non-differentiable. As a consequence, we use the mean square error (MSE) to calculate the difference between the label heatmap and the estimated heatmap instead of the error between the label point and the estimated point.

(2) $$\begin{array}{l} f{\left(x,y\right)}_{\text{Gaussian}}=Ae^{-\left(\frac{{\left(x-x_{c}\right)}^{2}}{2\sigma_{x}}+\frac{{\left(y-y_{c}\right)}^{2}}{2\sigma_{y}}\right)} \end{array}$$

In Formula (2), *A* is the amplitude that is fixed to 1, σ_*x*_ and σ_*y*_ are variances that we set to 3, and *x*_c_, and *y*_c_ are the central coordinates of the label landmark point.

Thirdly, the FCR method ignores the spatial information of the estimated points, while the HMP method obtains coordinate positions indirectly with quantitative errors. Therefore, we consider integral regression to calculate the coordinates of points from the heatmap (Nibali et al., 2018; Sun et al., 2018), called the heatmap integral regression (HIR) method. For one-dimensional data, the integral regression method uses SoftMax function to calculate the probability of each value and multiply it by the location coordinates of the value, as shown in Formula (3), which is termed the soft-argmax process. For each landmark point, we can estimate one heatmap, then we calculate the probability by SoftMax for each value in the heatmap and then sum by row and column. Subsequently, we multiply the sum result of each row and column by the location of the corresponding row and column and then accumulate the results of all rows and columns, respectively, to obtain the abscissa and ordinate of the estimated point. Finally, if the size of the heatmap is different from that of the input image, the coordinates need to be normalized to the input image. The whole process of the HIR method is shown in Figure 2B. Therefore, for each heatmap, we can estimate the two-dimensional coordinates of the landmark point by the HIR method, which can be proven as differentiable. We can use smooth L1 loss to calculate the error between the label landmark point and the estimated landmark point again as follows:

(3) $$\begin{array}{l} \sigma \left(z\right)=\overset{m}{\underset{i=1}{\sum }}\frac{e^{z_{i}}}{\overset{m}{\underset{j=1}{\sum }}e^{z_{j}}}i \end{array}$$

In Formula (3), σ(*z*) is the result of integral regression, *z* is the value of the one-dimensional data, *m* is the length of the data, and *i* and *j* are the locations of the value.

### Experimental Environment

**Figure 3:**
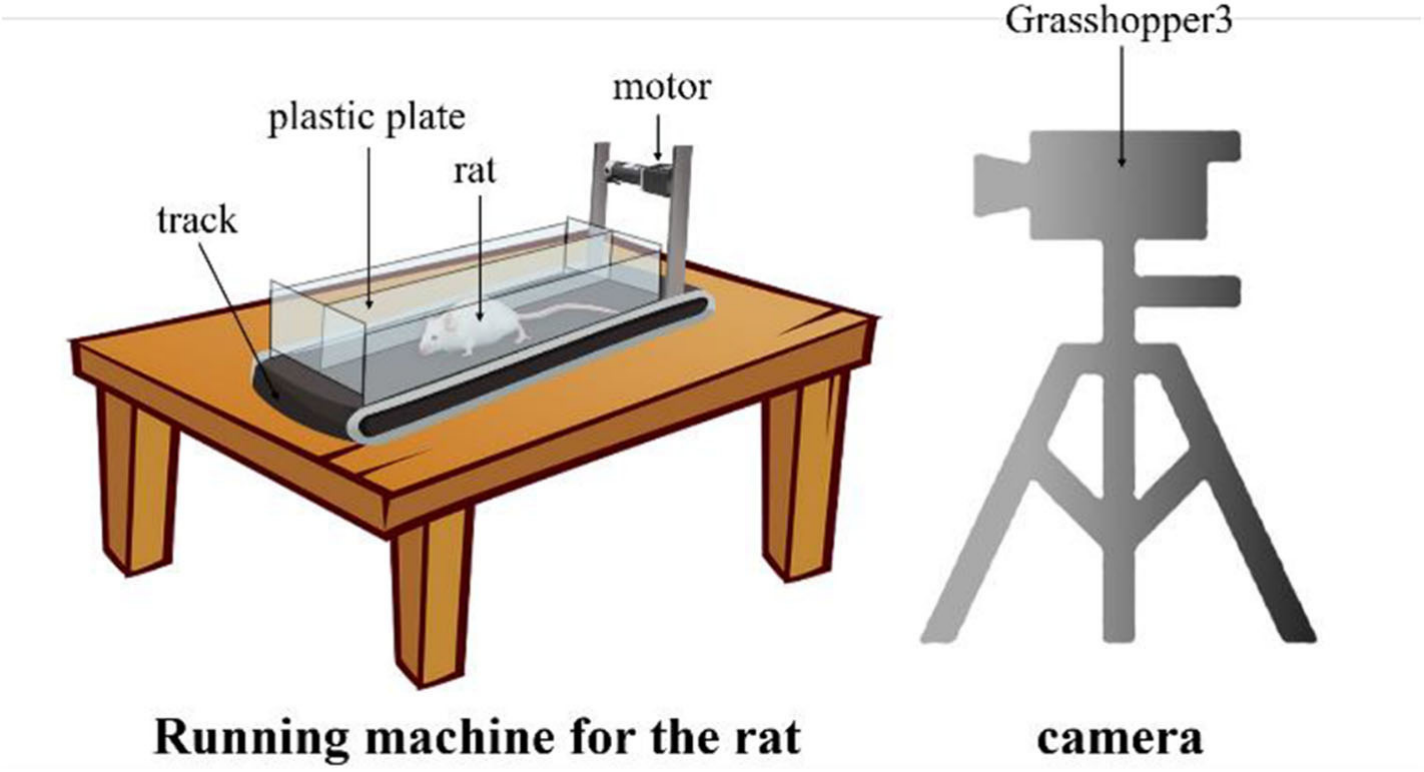
The rat motion observation device. The rat is placed on the special running machine for exercise and the high frame rat camera captures the motion from the side.

Since there is no publicly available dataset for rat landmark estimation, we collect the data ourselves. We build the rat motion observation device comprising a small running machine and a camera, as shown in Figure 3. The small running machine (length × width, 500 mm × 200 mm) is placed on the table. A 12V DC torque motor is used to drive the running machine track. In order to carry out the experiment and prevent the rat from running out of the range of the running machine, a 200-mm-high plastic plate is added around the running machine. At the same time, in order to prevent rats from turning and other behaviors that would influence the observations, a plastic plate is used in the middle of the track to separate the runway in a manner that is suitable for one-way movement of the rat. Since the motion of the rat's claws is very fast, it is very difficult to capture with an ordinary camera because of certain issues such as blur or smear. Therefore, we use the professional high frame rate camera Grasshopper3 and set the acquisition rate to 100 frames per second with a 1,056 × 720 resolution.

### Data Acquisition and Label

We selected a 3-week-old *Rattus norvegicus* to perform the landmark estimation experiment. The rat was placed on the running machine's runway and could only perform a one-way exercise. When the running machine is turned on, a high frame rate camera is used to capture the movement of the rat from the side. We then selected and eliminated the images of the rat's abnormal movement frame by frame and finally obtained a dataset of 1,613 normal movement images on the running machine.

**Figure 4:**
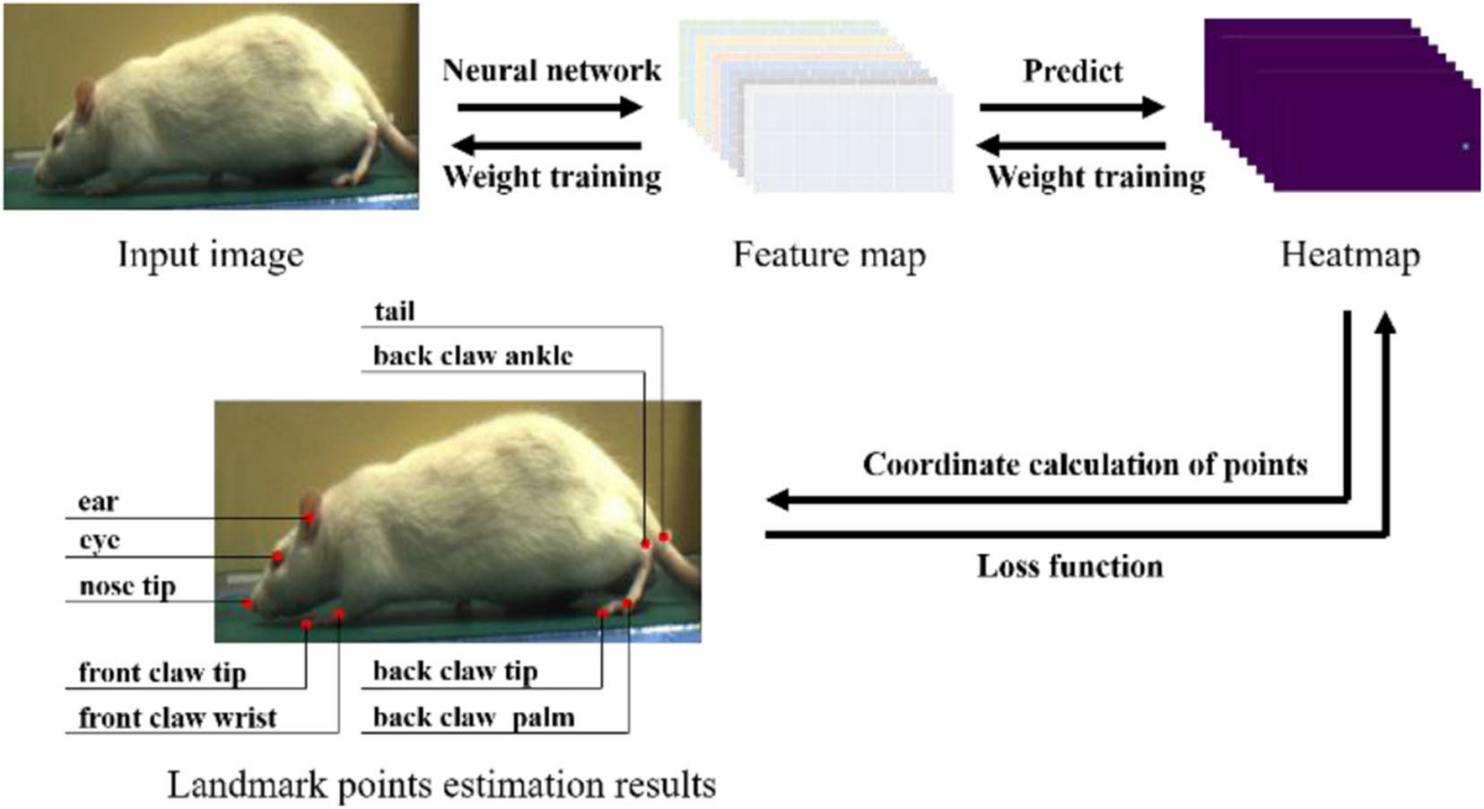
The experiment process of rat landmark point estimation. In the prediction process, the rat's image is input into the neural network for feature extraction, and the feature map obtained is used to predict the heatmap (taking the HMP method as an example). The coordinates of the rat landmark points are calculated from the heatmap. In the training process, the error between the label and the prediction coordinates is calculated by the loss function, and then gradient descent is used to reduce the training weight of the neural network.

After we obtained the dataset, it needed to be labeled according to the estimated landmark points. We selected nine landmark points for labeling and estimation. As shown in Figure 4, the landmark points include the nose tip, eye, ear, front claw wrist, front claw tip, back claw ankle, back claw palm, back claw tip, and tail. It is worth noting that we intended to label more landmark points, such as the knee and hip joints, but these landmark points are obscured by the rat's fur, and thus it is challenging to assign accurate labels to these. Three researchers participated in the labeling work, with each researcher randomly selecting images for labeling. Finally, a dataset of 1,613 images was obtained. In the process of neural network training, we randomly selected 80% of the dataset as the training set and 20% as the testing set, and each training result was averaged after multiple training.

### Model Training

As shown in Figure 4, in order to estimate the landmark points, it is necessary to train the robustness of the neural network model for feature extraction and prediction and then use an appropriate coordinate calculation method to calculate the coordinates of the landmark points in the image. Our methods are implemented by using PyTorch (Paszke et al., 2019) and ran on an Intel Core i5-6500 at 3.2 GHz desktop with a GeForce 980Ti GPU. All of the datasets used are manually labeled by ourselves. In addition, certain training techniques are utilized to improve the robustness of the network model.

Firstly, in the task of human pose estimation, the resolution of the input image is always resized into a square, e.g., 416 × 416. However, for the rat landmark point estimation task, the height and width of the rat are always unequal in the images, so we use images with unequal width and height as the input of the neural network, e.g., 512 × 256.

Secondly, the training data that we obtained by acquisition and labeling in the laboratory are limited. In order to improve the generalization ability of the model, we consider the random transformation of input data in the hue, saturation, value (HSV) space to increase the robustness of color and light transformation.

Thirdly, the two neural network structures that we designed possess many layers, which may cause the problem of gradient disappearance in the process of training, and this is difficult to converge. Therefore, we also use intermediate supervision to help deep network training and improve the estimation quality. As shown in Figure 1, each neural network structure has more than one estimation outputs. In the training process, we calculate the loss function of each estimated output for supervision learning. In the inference process, we only use the last output as the estimation of the landmark.

### Normalized Evaluation Criterion

During the experiment, we use CCN and CHN, two different neural network structures, to extract features and predict outputs. Moreover, we also use FCR, HMP, and HIR, three coordinate calculation methods, to obtain the coordinates of each landmark point. Therefore, an objective evaluation method is required for reasonable evaluation. It is subjective to directly calculate the pixel distance between the estimated coordinates and the label coordinates as the error for evaluation. Due to the different body shape or the different camera capture distance, the size of the rat displayed in the image also varied, and evaluation of the same pixel error for these different sized rats in the images should be different. For this reason, we refer to the evaluation criteria in the human pose estimation dataset MPII, named percentage of correct keypoints by a fraction of the head size (PCKh) (Andriluka et al., 2014). We then use the distance between the rat's nose and eye as the normalized denominator. Our normalized evaluation criteria are shown in Formula (4).

(4) $$\begin{array}{l} \text{error}\left(x_{i}^{\text{pred}},y_{i}^{\text{pred}}\right)=|\frac{\text{dis}\left(\left(x_{i}^{\text{pred}},y_{i}^{\text{pred}}\right),\left(x_{i}^{\text{label}},y_{i}^{\text{label}}\right)\right)}{\text{dis}\left(\left(x_{\text{nose}}^{\text{label}},y_{nose}^{\text{label}}\right),\left(x_{\text{eye}}^{\text{label}},y_{\text{eye}}^{\text{label}}\right)\right)}|-p \end{array}$$

In Formula (4), *i* is the *i*th estimated point and $\left(x_{i}^{\text{pred}},y_{i}^{\text{pred}}\right)$ are the predicted coordinates of the *i*th landmark point and are the labels. *p* is the Euclidean distance between two coordinates and *p* is the evaluation parameter that we set to 0.1. Therefore, the meaning of this formula is that, for the *i*th estimated point error, $\text{error}\left(x_{i}^{\text{pred}},y_{i}^{\text{pred}}\right)$, the Euclidean distance between the predicted coordinates and their corresponding label coordinates is divided by the Euclidean distance between the label coordinates of the nose and eye points and then subtracting an evaluation parameter, *p*.

When calculating the accuracy of the estimated points, the method in Formula (5) is used for statistics. If the error of the point is < 0, the estimation is considered to be accurate; otherwise, the estimation is considered to be incorrect. Finally, the accuracy of each landmark point in each method can be calculated as follows:

(5) $$\begin{array}{l} f\left(i\right)=\{\begin{array}{ll} 1 & \text{error}\left(x_{i}^{\text{pred}},y_{i}^{\text{pred}}\right)\leq 0,\text{correct}\\ 0 & \text{error}\left(x_{i}^{\text{pred}},y_{i}^{\text{pred}}\right)>0,\text{wrong} \end{array} \end{array}$$

## Results and Discussion

### Neural Network Structure Effect

From Table 1, we can analyze the effect of the network structure. In any coordinate calculation method, the accuracy of the CCN structure is greater than that of the CHN structure in terms of the overall average accuracy. CHN possesses certain advantages, such as the accuracy of certain landmark points, e.g., the tail, being better, although it has more network parameters, and the network floating-point operations (GFLOPs) are less due to using the hourglass network. In fact, although the hourglass network is commonly utilized in the field of human pose estimation, in our experiment, our CHN structure with the hourglass network is not superior to our CCN structure. The reason for this may be that the hourglass network loses too much information in the process of maximum pooling downsampling and nearest neighbor upsampling; more information may be retained if the network channel is added in the hourglass structure.

**Table 1:** Estimation accuracy comparison on the test set with normalized evaluation criteria (*p* = 0.1).

**Network structure** **and calculation** **method**	**No. of** **parameters**	**GFLOPs**	**Training** **time (h)**	**Testing** **time (s)**	**Nose** **tip (%)**	**Eye** **(%)**	**Ear** **(%)**	**Front claw** **wrist (%)**	**Front claw** **tip (%)**	**Back claw** **ankle (%)**	**Back claw** **palm (%)**	**Back claw** **tip (%)**	**Tail** **(%)**	**Total** **(%)**
CCN+FCR	108.5M	59.78	9.62	59.37	64.4	74.3	62.5	40.4	41.4	54.5	52.9	53.9	34.8	53.2
CCN+HMP	72.5M	59.76	9.72	59.40	59.9	60.2	50.9	40.9	54.0	53.6	53.2	54.6	52.6	53.3
CCN+HIR	72.5M	59.76	9.74	60.40	88.2	92.7	82.5	61.3	76.2	76.7	75.6	76.4	45.5	75.0
CHN+FCR	103.6M	45.25	7.92	45.60	61.1	63.5	56.3	36.3	40.9	48.4	47.3	45.7	35.1	48.3
CHN+HMP	85.6M	45.24	7.98	45.94	53.5	56.3	51.6	36,7	47.0	53.5	48.0	50.7	48.4	49.5
CHN+HIR	85.6M	45.24	7.92	44.90	79.7	90.0	76.2	56.3	69.1	73.3	74.4	76.6	62.5	73.1

The length of the video in the training session is 10 s.

*CCN, cascade convolution network; CHN, cascade hourglass network; FCR, fully connected regression; HMP, heatmap maximum position; HIR, heatmap integral regression; GFLOPs, giga floating-point operations*.

**Figure 5:**
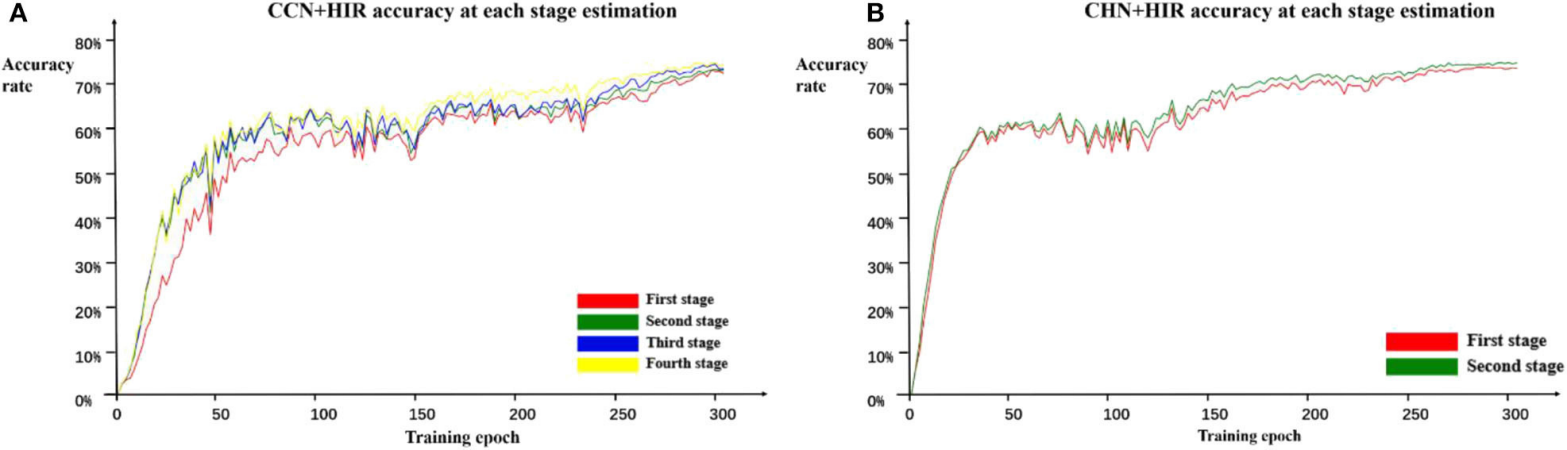
Estimation accuracy of each stage in the CCN+HIR **(A)** and CHN+HIR **(B)** approaches. During the training, we record the test results of each stage to verify the effectiveness of the cascade structure.

In addition, because each stage of the cascade network designed in this paper can estimate the landmark points independently and in order to further verify the role of the cascade structure, we calculate the accuracy on the test set every two epochs of training and record the accuracy of each stage during training. The estimation accuracy results of the CCN and CHN network structures using the HIR method are shown in Figure 5. For CCN, in the early epochs of training, the accuracy of the second-stage estimation is significantly improved compared with the first-stage estimation, but the accuracy of the second-stage estimation to the fourth-stage estimation does not change. Moreover, as the number of training epochs increases, the accuracy difference between the first-stage and the second-stage estimation gradually decreases and that from the second-stage estimation to the fourth-stage estimation begins to appear. For CHN, although it is only designed as a two-stage cascade structure, from the whole training process, the accuracy of the second-stage estimation is indeed higher than that of the first-stage estimation. Therefore, it can be found that, irrespective of whether it is the CCN or CHN, the multistage cascade structure can substantially improve the accuracy of the estimated points. Although only the results using the HIR method are given here, these are consistent for the other two coordinate calculation methods.

### Coordinate Calculation Method Effect

**Figure 6:**
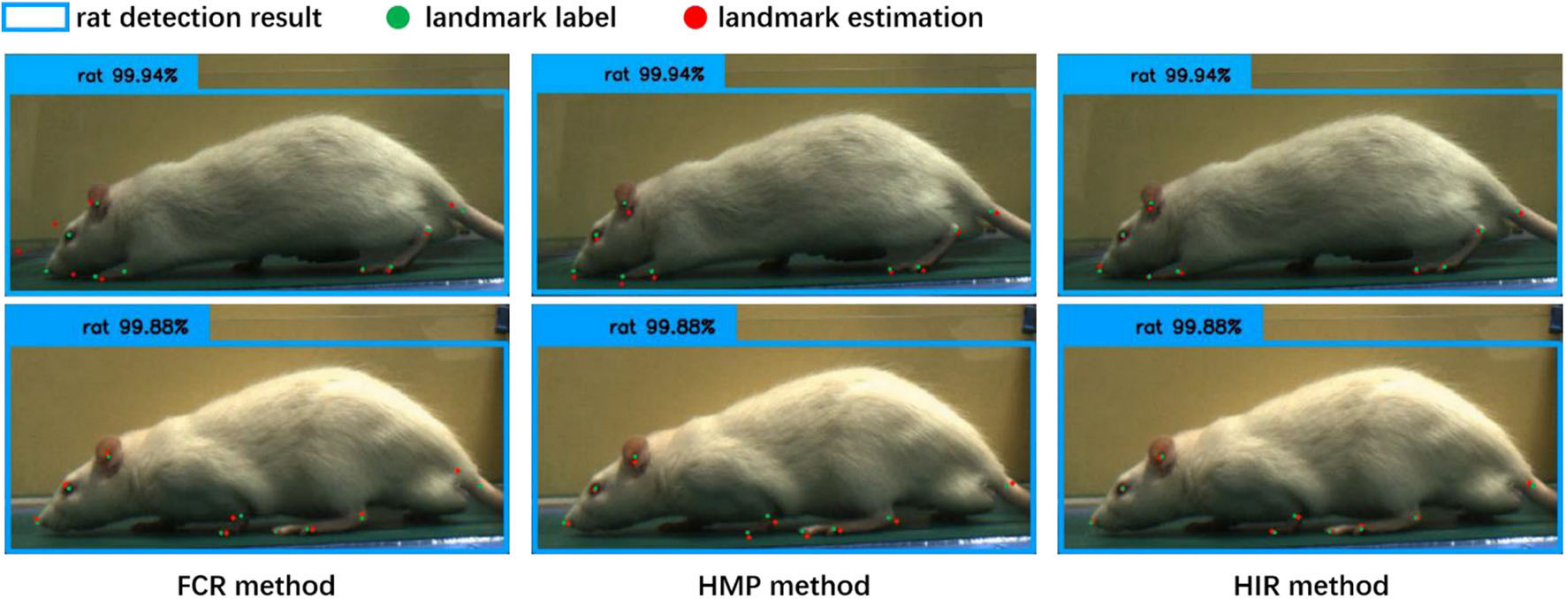
The results of the landmark point estimation with three coordinate calculation methods (cascade convolution network, CCN). The *blue bounding box* is the detection result by rat behavior observation [11], the *green point* is the label of the landmarks, and the *red point* is the estimation result of the landmarks. In the fully connected regression (*FCR*) method, only a part of the estimation points can be close to the label, and the estimation error of the landmark points is large. In the heatmap maximum position (*HMP*) method, each estimation point is close to the label coordinates, but with some estimation errors. In the heatmap integral regression (*HIR*) method, each estimation point, and label coordinate are closely similar or even overlapped; the landmark point estimation results are accurate.

From Table 1, regardless of whether it is the CCN or the CHN, the overall accuracy of the FCR method in calculating the coordinates of the landmark points is the worst, and the fully connected layer is added to increase the network parameters. Specifically, however, the FCR method has a higher accuracy for some landmark points with little change, such as the nose, eyes, and ears points, while for some frequently changed points such as the claw wrist or tip has lower accuracy. From the results of the image landmark point estimation, shown in Figure 6, the FCR method is good in the estimation of landmark points in the back claw, while the estimation error of the other landmark points is large. Based on the analysis of Table 1 and Figure 6, we conclude that there is serious overfitting in the points with little changes, such as the eyes, nose, and ears, while there is underfitting in the front claw landmark points.

Table 1 also shows that the HMP method is only slightly better than the FCR method overall. However, for each of the two neural network structures, the estimation accuracy of each landmark point by the HMP method is relatively average, which may offer certain spatial generalization ability. In addition, according to the results of the image landmark point estimation shown in Figure 6, the HMP method is much better than the FCR, and every landmark point can be estimated to the corresponding position and move with the rat's motion. Careful observation, however, shows that there is a small distance deviation between the estimated point coordinates and the real coordinates in some estimation points; using our strict normalization evaluation criterion, these points with deviations cannot be considered as correct. We conclude that this mainly comes from the quantization error resultant from the unequal size of the heatmap and the input image because of the downsampling in the basic feature extraction network.

Regarding the HIR method, from Table 1, irrespective of whether it is the CCN or the CHN, it achieves higher accuracy than the other two methods without increases in any network parameters or GFLOPs. On each item, the HIR method not only has a far higher than average accuracy for the little-changing landmark points, such as the nose and the eyes, but also achieves accurate estimation for the frequently changing points, such as moving claws. Similarly, from the results in Figure 6, the HIR method eliminates some quantization errors through regression, and the estimation results are excellent. Indeed, each landmark point can accurately follow the rat's movement.

Finally, combining the neural network structures and coordinate calculation methods, it is demonstrated that using the CCN structure and the HIR method constitutes the optimal approach for the task of rat landmark point estimation and achieves 75% accuracy in the test set.

### Feature Map Size Effect

In the part of the neural network structure, we introduce the basic feature extraction network. The feature map size is one-eighth of the input image size, which leads to the quantization error in the HMP method. In order to elucidate the effect of quantization error, we modify the basic feature extraction network to make the size of the feature map larger. Specifically, based on the original basic feature extraction network, we delete two consecutive convolution layers and one downsampling layer so that the size of the feature map is one-fourth of the input image. In this case, we still use CCN and CHN, two network structures, as well as FCR, HMP, and HIR, three coordinate calculation methods, to retrain the network model. The results on the test set are presented in Table 2.

**Table 2:** Estimation accuracy comparison on the test set with modified basic feature extraction network (*p* = 0.1).

**Network structure and calculation method**	**No. of parameters**	**GFLOPs**	**Training time (h)**	**Testing time (s)**	**Nose tip (%)**	**Eye (%)**	**Ear (%)**	**Front claw wrist (%)**	**Front claw tip (%)**	**Back claw ankle (%)**	**Back claw palm (%)**	**Back claw tip (%)**	**Tail (%)**	**Total (%)**
CCN+FCR	189.5M	107.85	14.17	50.10	47.9	58.0	57.5	30.4	27.9	39.5	34.7	33.5	10.7	37.8
CCN+HMP	45.4M	107.78	13.89	50.91	85.4	90.9	81.9	65.2	76.2	81.4	80.9	82.7	80.2	80.5
CCN+HIR	45.4M	107.78	14.05	49.05	90.4	95.0	85.0	64.5	80.7	80.9	74.9	86.7	51.0	78.8
CHN+FCR	130.6M	49.75	10.38	43.33	28.9	41.6	38.4	20.6	21.4	25.4	21.8	19.4	7.8	25.0
CHN+HMP	58.6M	49.71	10.25	44.53	79.2	85.5	76.9	58.0	70.8	73.3	72.3	74.7	69.2	73.3
CHN+HIR	58.6M	49.71	10.35	43.22	85.1	93.7	78.0	54.6	75.4	77.9	73.7	78.6	69.4	76.2

The length of the video in the training session is 10 s.

*CN, cascade convolution network; CHN, cascade hourglass network; FCR, fully connected regression; HMP, heatmap maximum position; HIR, heatmap integral regression; GFLOPs, giga floating-point operations*.

By comparing Table 1 with Table 2, concerning the neural network structure, the estimation accuracy of the CCN is still better than that of the CHN when the size of the feature map is larger. However, the larger feature map results in more floating-point calculations, especially in the case of the CCN structure which uses a large number of convolution layers, while the CHN structure increases slightly due to further sampling in the hourglass network.

Regarding the coordinate calculation method in both the CCN and CHN structures, there are many different results. Firstly, in the FCR method, with the increase of the feature map size, the estimation accuracy has been greatly decreased. The reason for this is that the increased size of the feature map leads to the increase of neurons in the fully connected layer, which makes the regression calculation task more complex and more prone to overfitting, finally making estimation of the landmark points unstable. Secondly, in the HMP method, the accuracy of its estimation has increased significantly. The reason for this is that, in the original size of the feature map, the error mainly derives from the quantization error, which cannot be recognized by the strict normalization evaluation criterion. With the increased size of the feature map, however, part of the quantization error is eliminated and more estimated landmark points are recognized. Thirdly, in the HIR method, the overall accuracy is only slightly improved compared to that shown in Table 1. Moreover, when using the CCN structure, accuracy has been surpassed by the HMP method, and it can be found that the main problem is that the accuracy of the tail landmark point is markedly lower than the average. When using the CHN structure, the HIR method is still better than the HMP. From these results, it is shown that the HMP method possesses strong spatial generalization ability and the estimation accuracy for each point is relatively average, whereas the HIR method will sacrifice part of the spatial ability in the regression process, resulting in the reduction of accuracy at certain points. Furthermore, since the CHN has more sampling processes and loses some spatial characteristics of the landmark points, its accuracy is lower than that of the CCN.

Finally, although the accuracy of the rat landmark point estimation will increase with the increase of the scale of the feature map, additional computation will be introduced. Based on the comprehensive analysis, it is demonstrated that the CCN+HIR approach is the best for small feature maps and that the CHN+HIR approach can be considered for large feature maps.

### Quantification of Rat Joint Motion

**Figure 7:**
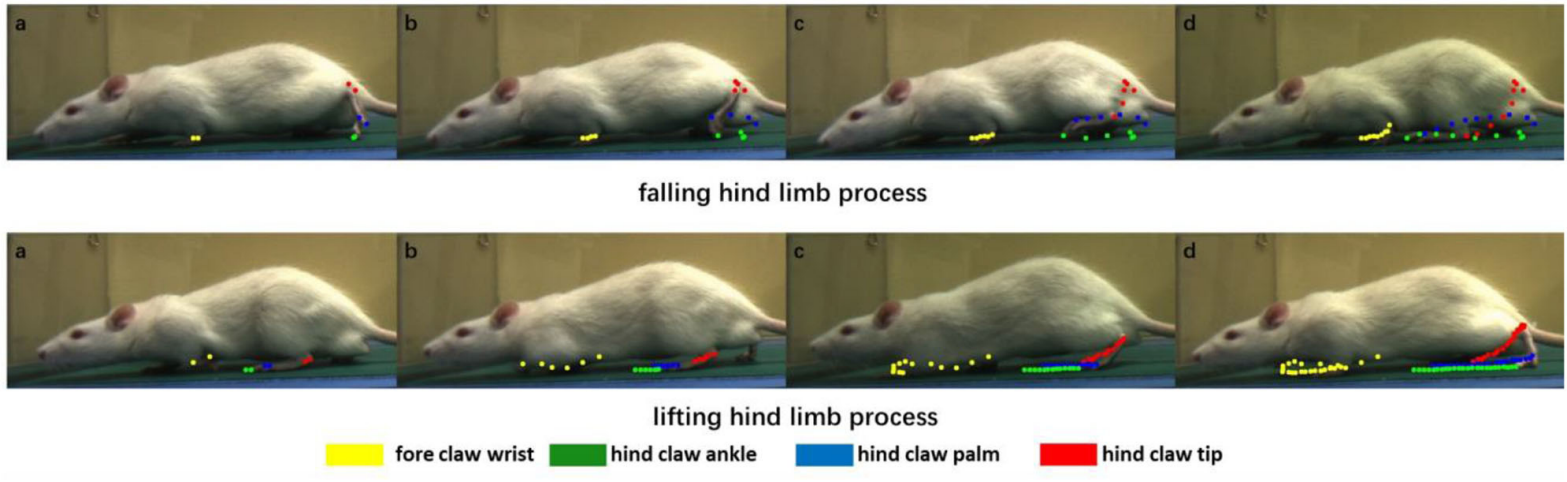
Dynamic motion of certain joints in the rat. We divide the rat gait motion periodically into two processes to show the result, which are the falling hind limb process and the lifting hind limb process. Each color corresponds to one joint: *yellow, green, blue*, and *red* correspond to the fore claw wrist, hind claw ankle, hind claw palm, and hind claw tip, respectively. In the two processes, the joint coordinates of each frame are recorded and displayed in the images. *From left to right*, these images show the joint motion so as to quantify the clear joint motion track.

**Figure 8:**
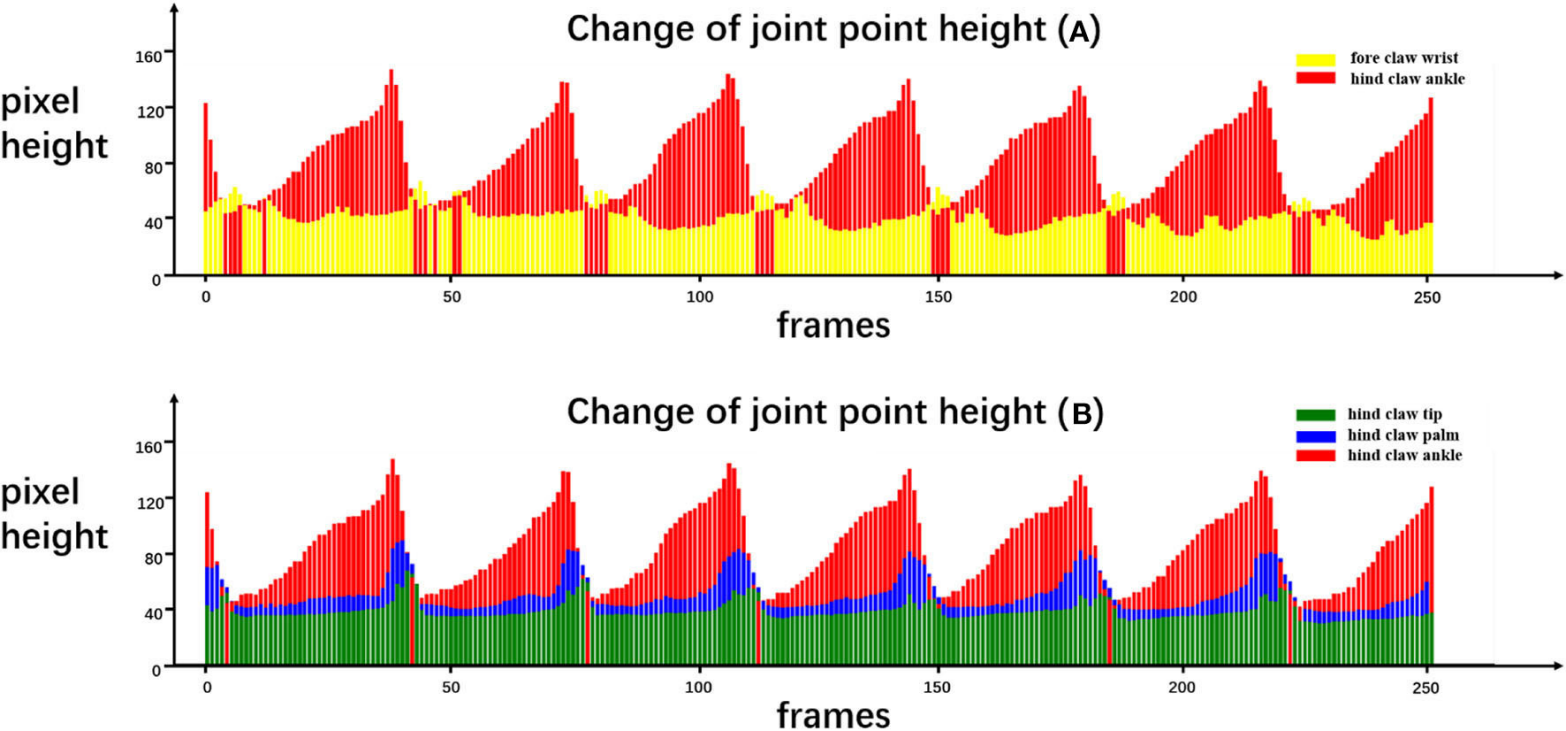
Quantitative results of certain joint movements. We recorded the movement of the rat in our running machine experiment for a period of time and estimated the coordinates of the landmark joint points at each frame. We drew the height of the rat joint points in each frame as the quantitative results to analyze the rat's behavior. In the change of joint point height **(A)**, *yellow* and *red* correspond to the height of the fore claw wrist joint point and the hind claw ankle joint point, respectively. In the change of joint point height **(B)**, *green, blue*, and *red* correspond to the height of the hind paw tip, hind paw palm, and hind paw ankle, respectively.

The purpose of our rat landmark point estimation was to track and quantify rat joint motion in order to assist research in medicine, neuroscience, and other fields. Here, we track and quantify some of the rat joints using the video and analyze the rat gait movement as an example. Because many joints of the rat are obscured by the fur, here, we only select fore claw wrist, hind claw ankle, hind claw palm, and hind claw tip, four joint points, and use our rat landmark point estimation CCN+HIR approach to track and quantify them. The dynamic tracking results are presented in Figure 7. The quantitative results of each joint motion in a period of time are shown in Figure 8.

Figure 7 includes several images of the rat in the process of crawling, and the positions of the joint are estimated. From left to right, each joint position of the rat in the past few frames were recorded through different color points. It can be seen that, in the falling hind limb process, the movement distance of the hind limb is significantly longer than that of the forelimb, whereas in the lifting hind limb process, the movement distance of the forelimb is longer. At the same time, according to the points of different colors in Figure 7, it is possible to draw the motion trajectory of each joint. For the trajectory of three joints in the hind limb of the rat, there are different degrees of intersection in the longitudinal and transverse positions in the process of movement.

From Figure 8, certain quantitative information can be discerned that cannot be obtained only by the naked eyes. For example, Figure 8A quantifies the height of the front claw wrist and the hind claw ankle, and it can be seen that the lifting speed of the hind claw is slower than the falling speed, while the front claw has no obvious difference. Moreover, each time that the front claw is lifted, two obvious peaks exist in the process of the fore claw wrist forward protrusion and the moment when the hind claw ankle point moves to the lowest position corresponding to the first peak of the front claw wrist point movement. Figure 8B quantifies the height of the hind claw ankle, hind claw palm, and hind claw tip. It can be seen that the movement of the hind claw is similar, but the timing of lifting is slightly different. Specifically, the hind claw ankle joint is first lifted, followed by the palm joint and the tip joint, and when the ankle joint lands, the palm joint and the tip have not yet landed, so we can find that, in some frames, the height of the ankle joint will be lower than those of the other two joints. On this basis, we can utilize the pre-trained model to analyze the gait movement of the spinal cord injury (SCI) rat with electrical epidural stimulation (EES) surgery (Moraud et al., 2016), which provides a markerless and low-cost observation method for further research.

## Conclusion

In this paper, we focus on the estimation of rat landmark points and the quantification of joint motion without invasive sensors or markers. The four-stage convolution cascade network (CCN) and the two-stage cascade hourglass network (CHN) are designed to extract features. Three coordinate calculation methods—the FCR method, HMP method, and the HIR method—are used to calculate the coordinates of the landmark points. We also propose a normalized evaluation criterion to evaluate these different network structures and coordinate calculation methods. It is demonstrated that the CCN structure achieves higher accuracy, but the CHN structure requires less computation. After comparing these network structures and coordinate calculation methods in detail, the CCN+HIR approach is shown to be the best for the small-sized feature map and the CHN+HIR approach can be considered when the feature map size is increased. Finally, we use our landmark point estimation approach to quantify joint motion in the process of rat movement. In the future, we will investigate whether our rat landmark estimation approach can calculate the angular velocity and angular acceleration of the joints of SCI rats to evaluate the rehabilitation effect, which could constitute a useful protocol for clinical applications in humans.

## Data Availability Statement

The raw data supporting the conclusions of this article will be made available by the authors, without undue reservation.

## Ethics Statement

The animal study was reviewed and approved by Nankai University animal ethics committee.

## Author Contributions

TJ and FD: conceptualization, writing—review, and editing. TJ and SY: methodology. ZY and YL: validation and supervision. XC and QY: formal analysis and investigation. TJ and ZY: data curation. TJ: writing—original draft preparation. SY and FJ: visualization. FD: project administration and funding acquisition. All authors have read and agreed to the published version of the manuscript.

## Conflict of Interest

The authors declare that the research was conducted in the absence of any commercial or financial relationships that could be construed as a potential conflict of interest.
